# Influence of insulin-like growth factor I overexpression via recombinant adeno-associated vector gene transfer upon the biological activities and differentiation potential of human bone marrow-derived mesenchymal stem cells

**DOI:** 10.1186/scrt491

**Published:** 2014-08-27

**Authors:** Janina Frisch, Jagadeesh Kumar Venkatesan, Ana Rey-Rico, Gertrud Schmitt, Henning Madry, Magali Cucchiarini

**Affiliations:** Center of Experimental Orthopaedics, Saarland University Medical Center, Kirrbergerstr. Bldg 37, D-66421 Homburg/Saar, Germany; Department of Orthopaedic Surgery, Saarland University Medical Center, Kirrbergerstr. Bldg 37, D-66421 Homburg/Saar, Germany

## Abstract

**Introduction:**

The transplantation of genetically modified progenitor cells such as bone marrow-derived mesenchymal stem cells (MSCs) is an attractive strategy to improve the natural healing of articular cartilage defects. In the present study, we examined the potential benefits of sustained overexpression of the mitogenic and pro-anabolic insulin-like growth factor I (IGF-I) via gene transfer upon the biological activities of human MSCs (hMSCs).

**Methods:**

Recombinant adeno-associated vectors (rAAV) were used to deliver a human IGF-I coding sequence in undifferentiated and chondrogenically-induced primary hMSCs in order to determine the efficacy and duration of transgene expression and the subsequent effects of the genetic modification upon the chondrogenic *versus* osteogenic differentiation profiles of the cells relative to control (*lacZ*) treatment after 21 days *in vitro*.

**Results:**

Significant and prolonged expression of IGF-I was evidenced in undifferentiated and most importantly in chondrogenically-induced hMSCs transduced with the candidate rAAV-hIGF-I vector for up to 21 days, leading to enhanced proliferative, biosynthetic, and chondrogenic activities compared with rAAV-*lacZ* treatment. Overexpression of IGF-I as achieved in the conditions applied here also increased the expression of hypertrophic and osteogenic markers in the treated cells.

**Conclusions:**

These results suggest that a tight regulation of rAAV expression may be necessary for further translation of the approach in clinically relevant animal models *in vivo*. However, the current findings support the concept of using this type of vector as an effective tool to treat articular cartilage defects via gene- and stem cell-based procedures.

## Introduction

Injured adult articular cartilage, the tissue that allows for smooth gliding and weight bearing on articulating surfaces, does not heal effectively by itself [ 1]. Due to its aneural and avascular nature and in the absence of lymphatic drainage, the articular cartilage does not have access to reparative cells that are potentially brought into nearly all other tissues in response to injury. Lesions such as those resulting from acute trauma or degenerative osteoarthritis become persistent and progress over time [ 1, 2]. Hyaline cartilage is formed by chondrocytes surrounded by a dense network of extracellular matrix composed of proteoglycans bound to 70 to 80% water and type II collagen and other molecules (type IX, type XI, type VI, and type XIV collagen, cartilage oligomeric matrix protein, link protein, decorin, fibromodulin, fibronectin, tenascin). Despite the availability of various pharmacological treatments and surgical interventions, reproduction of the native organization and activities in injured cartilage remains problematic [ 3– 6] because such options promote mostly the appearance of a fibrocartilaginous repair tissue consisting of type I collagen that does not integrate well with the surrounding cartilage and poorly withstands mechanical stress [ 1, 6– 8].

Large efforts have been made to overcome these difficulties, and the concept of treating cartilage lesions by stem cell-based therapy became an important focus of experimental and clinical research for cartilage repair – taking advantage of the chondrogenic differentiation potential and regenerative properties of such cells, including bone marrow-derived mesenchymal stem cells (MSCs) [ 9– 13]. Important limitations still hinder the use of MSCs in patients, including the large amounts of cells required for application *in vivo* and the age-related decline in lifespan, proliferation, and potency [ 14– 17]. Gene delivery approaches offer strong tools to optimize the use of human bone marrow-derived mesenchymal stem cells (hMSCs) for cartilage repair purposes. Various therapeutic candidate sequences have been reported for their effects upon the chondrogenic differentiation of such cells, among which are cartilage oligomeric matrix protein [ 18], transforming growth factor beta (TGFβ) [ 19– 21], bone morphogenetic proteins [ 21– 23], basic fibroblast growth factor (FGF-2) [ 24], Indian hedgehog [ 21], human telomerase alone [ 25, 26] or combined with a small interfering RNA against p53 [ 27], the specific transcription factors of the SOX family alone [ 28– 33] or combined with an anti-Runx2/Cbfa1 small interfering RNA [ 34], or the zinc-finger protein 145 [ 35]. Most of these studies, however, focused on the use of gene transfer vectors with relatively low or short-term efficiencies (nonviral vectors, adenoviral vectors) [ 18, 19, 21– 23, 28– 31, 33, 34] or on constructs carrying the risk of insertional mutagenesis (retroviral vectors, lentiviral vectors) [ 25– 27, 35].

Recombinant adeno-associated virus (rAAV) vectors emerged instead as more advantageous gene vehicles because they are less toxic and immunogenic due to complete removal of the adeno-associated viral vector coding sequences while allowing for very high and persistent levels of transgene expression in hMSCs by maintenance of the sequences delivered mostly under the form of stable episomes, without impairment of the differentiation potential [ 20, 24, 32]. Genetic modification of hMSCs via rAAV has so far been performed to deliver various therapeutic candidates including TGFβ [ 20], FGF-2 [ 24], and SOX9 [ 32], but little is known about the effects of applying insulin-like growth factor I (IGF-I) via rAAV in this clinically relevant population of regenerative cells. In the present study, we also focused on this particular growth factor in light of our previous work showing the benefits of overexpressing IGF-I via rAAV upon the remodeling of human osteoarthritic cartilage by activation of the anabolic and proliferative processes in damaged chondrocytes *in situ* [ 36]. Our results demonstrate – for the first time, to our best knowledge – that chondrogenic differentiation of hMSCs can be enhanced by treatment with IGF-I, specifically when overexpressing the factor via rAAV gene transfer possibly due to the high and prolonged levels of transgene expression achieved with this class of vector compared with other less efficient gene delivery systems. Yet treatment with the current IGF-I candidate vector also led to an increased expression of hypertrophic and osteogenic markers, so tight regulation of IGF-I expression via rAAV will be critical to achieve an optimized stem cell-based, rAAV-human insulin-like growth factor I (hIGF-I)-mediated approach to treat articular cartilage defects *in vivo*.

## Methods

### Reagents

Reagents were obtained from Sigma (Munich, Germany) unless otherwise indicated. Recombinant FGF-2 and TGFβ3 were purchased from Peprotech (Hamburg, Germany). The dimethylmethylene blue dye was from Serva (Heidelberg, Germany). The anti-IGF-I (AF-291-NA) antibody was from R&D Systems (Wiesbaden-Nordenstadt, Germany), the anti-type-II collagen (II-II6B3) antibody from the NIH Hybridoma Bank (University of Iowa, Ames, IO, USA), the anti-type-I (AF-5610) antibody from Acris (Hiddenhausen, Germany), the anti-bromodeoxyuridine (BrdU; BU-33) and anti-type X collagen (COL-10) antibodies from Sigma, and the anti-SOX9 (C-20), anti-CD34 (C-18), anti-CD71 (C-20), and anti-CD105 (T-20) antibodies from Santa Cruz Biotechnology (Heidelberg, Germany). Biotinylated secondary antibodies and ABC reagent were obtained from Vector Laboratories (Alexis Deutschland GmbH, Grünberg, Germany). The IGF-I enzyme-linked immunosorbent assay (ELISA) (hIGF-I Quantikine ELISA) was from R&D Systems, the type II and type I collagen ELISAs (Arthrogen-CIA Capture ELISA Kit) from Chondrex (Redmond, WA, USA), and the type X collagen ELISA from Antibodies-online GmbH (Aachen, Germany). The Cell Proliferation reagent WST-1 was from Roche Applied Science (Mannheim, Germany) and the alkaline phosphatase (ALP) staining kit was from Sigma.

### Cell culture

Bone marrow aspirates (~15 ml) were obtained from the distal femurs of patients undergoing total knee arthroplasty (*n* = 26). The study was approved by the Ethics Committee of the Saarland Physicians Council. All patients provided informed consent before inclusion in the study and all procedures were in accordance with the Helsinki Declaration. The hMSCs were isolated and expanded in culture using standard protocols [ 24, 32]. Briefly, aspirates were washed in Dulbecco’s modified Eagle’s medium (DMEM), centrifuged, and the pellet was resuspended in Red Blood Cell Lysing Buffer (Sigma) in DMEM (1:1). The resulting fraction was washed, pelleted, and resuspended in DMEM containing 10% fetal bovine serum with penicillin (100 U/ml)/streptomycin (100 μl/ml) (growth medium). Cells were plated in T75 flasks and maintained at 37°C in a humidified atmosphere with 5% carbon dioxide. The medium was exchanged after 24 hours and every 2 to 3 days thereafter using growth medium with recombinant FGF-2 (1 ng/ml). The cells were detached and replated for further experiments at appropriate densities. The hMSCs were analyzed with flow cytometry for expression of stem-cell surface markers (CD71^+^, CD105^+^, CD34^−^). All experiments were performed with cells at no more than passage two.

### Plasmids and recombinant adeno-associated virus vectors

The constructs were all derived from the same parental adeno-associated vector-2 genomic clone, pSSV9 [ 37, 38]. rAAV-*lacZ* carries the *lacZ* gene encoding *Escherichia coli* β-galactosidase and rAAV-hIGF-I carries a 536 base pair hIGF-I cDNA fragment [ 36], both under the control of the cytomegalovirus immediate-early promoter [ 24, 32, 36]. rAAV vectors were packaged as conventional (not self-complementary) vectors in the 293 adenovirus-transformed embryonic kidney cell line, using Adenovirus 5 to provide helper functions in combination with the pAd8 helper plasmid as described previously [ 24, 32, 36]. Purification, dialysis, and titration of the vectors by real-time polymerase chain reaction (PCR) were performed as described previously [ 24, 32, 36], averaging 10^10^ transgene copies/ml (ratio virus particles to functional vectors = 500/1).

### Recombinant adeno-associated virus-mediated gene transfer

Monolayer cultures of undifferentiated hMSCs (2 × 10^4^ cells) were transduced with rAAV (20 μl vector; that is, 4 × 10^5^ functional recombinant viral particles or multiplicity of infection (MOI) = 20) and kept in growth medium for up to 21 days [ 24, 32]. The hMSC aggregate cultures (2 × 10^5^ cells) were prepared and kept in defined chondrogenic medium (high-glucose DMEM 4.5 g/l, penicillin/streptomycin, 6.25 μg/ml insulin, 6.25 μg/ml transferrin, 6.25 μg/ml selenous acid, 5.35 μg/ml linoleic acid, 1.25 μg/ml bovine serum albumin, 1 mM sodium pyruvate, 37.5 μg/ml ascorbate 2-phosphate, 10^−7^ M dexamethasone, 10 ng/ml TGFβ3) for transduction (or not) with rAAV (40 μl vector; that is, 8 × 10^5^ functional recombinant viral particles or MOI = 4) over a period of 21 days [ 24, 32]. For osteogenic and adipogenic differentiation, hMSCs in monolayer cultures (10^5^ cells) were transduced with rAAV (40 μl vector; that is, 8 × 10^5^ functional recombinant viral particles or MOI = 8) and induced toward osteogenic differentiation using the StemPro Osteogenesis Differentiation kit or toward adipogenic differentiation using the StemPro Adipogenesis Differentiation kit (both from Life Technologies GmbH, Darmstadt, Germany) [ 32].

### Transgene expression

To assess IGF-I secretion, samples were washed twice and placed for 24 hours in serum-free medium. Supernatants were collected at the denoted time points and centrifuged to remove debris, and IGF-I production was measured by ELISA [ 36]. Quantitative measurements were performed on a GENios spectrophotometer/fluorometer (Tecan, Crailsheim, Germany). Transgene expression was also monitored by immunocytochemical and immunohistochemical analyses using a specific primary antibody [ 24, 32, 36].

### Biochemical assays

Cultures were harvested and aggregates were digested with papain [ 24, 32]. Cell proliferation was assessed with the Cell Proliferation reagent WST-1, with optical density proportional to the cell numbers [ 24, 32, 36], and by immunolabeling following BrdU incorporation (3 μg/ml for 24 hours) [ 36]. The DNA and proteoglycan contents were determined with a fluorimetric assay using Hoechst 22358 and by binding to dimethylmethylene blue dye, respectively [ 24, 32, 36]. Analysis of the type II, type I, and type X collagen contents was performed by respective ELISAs [ 24, 32, 36]. Data were normalized to total cellular proteins using a protein assay (Pierce Thermo Scientific, Fisher Scientific, Schwerte, Germany). All measurements were performed on a GENios spectrophotometer/fluorometer (Tecan).

### Histological, immunocytochemical, and immunohistochemical analyses

Monolayer and aggregate cultures were harvested and fixed in 4% formalin. Aggregates were further dehydrated in graded alcohols, embedded in paraffin, and sectioned (3 μm). Sections were stained with hematoxylin and eosin (cellularity), toluidine blue (matrix proteoglycans), and alizarin red (matrix mineralization) as described previously [ 24, 32]. Expression of SOX9, type II, type I, and type X collagen was detected by immunohistochemistry using specific primary antibodies, biotinylated secondary antibodies, and the ABC method with diaminobenzidine as the chromogen [ 24, 32]. Samples were also tested for transgene (IGF-I) expression and BrdU incorporation using specific primary antibodies. To control for secondary immunoglobulins, sections were processed with omission of the primary antibody. Osteogenically induced cultures were stained for ALP activity and adipogenically differentiated cultures were stained with Oil Red O to detect intracellular lipid droplets [ 32]. Samples were examined under light microscopy (Olympus BX 45; Olympus, Hamburg, Germany).

### Histomorphometry

The transduction efficiencies, the percentage of cells positive for BrdU uptake and for SOX9 expression, the cell densities on hematoxylin and eosin-stained sections, the aggregate diameters, the intensities of toluidine blue and alizarin red staining and those of type II, type I, and type X collagen immunostaining (all in pixels per standardized area) were measured at three random standardized sites or using 10 serial histologic and immunohistochemical sections for each parameter, test, and replicate condition using the SIS analySIS program (Olympus), Adobe Photoshop (Adobe Systems, Unterschleissheim, Germany), and Scion Image (Scion Corporation, Frederick, MD, USA) [ 24, 32]. The percentages of areas stained for ALP or Oil Red O were calculated as being the ratios of positively stained surface to the total surface evaluated [ 32]. The ALP and Oil red O staining intensities were measured in pixels per standardized area [ 32].

### Real-time reverse transcription polymerase chain reaction analyses

Total cellular RNA was extracted from the cultures using the RNeasy Protect Mini Kit with an on-column RNase-free DNase treatment (Qiagen, Hilden, Germany). RNA was eluted in 30 μl RNase-free water. Reverse transcription was carried out with 8 μl eluate using the 1st Strand cDNA Synthesis kit for RT-PCR (AMV; Roche Applied Science). An aliquot of the cDNA product (2 μl) was amplified with real-time PCR using the Brilliant SYBR Green QPCR Master Mix (Stratagene, Agilent Technologies, Waldbronn, Germany) on an Mx3000P QPCR operator system (Stratagene) as follows: initial incubation (95°C, 10 minutes), amplification for 55 cycles (denaturation at 95°C, 30 seconds; annealing at 55°C, 1 minute; extension at 72°C, 30 seconds), denaturation (95°C, 1 minute), and final incubation (55°C, 30 seconds). The primers (Invitrogen, Darmstadt, Germany) used were SOX9 (chondrogenic marker) (forward, 5′-ACACACAGCTCACTCGACCTTG-3′; reverse, 5′-GGGAATTCTGGTTGGTCCTCT-3′), type II collagen (COL2A1; chondrogenic marker) (forward, 5′-GGACTTTTCTCCCCTCTCT-3′; reverse, 5′-GACCCGAAGGTCTTACAGGA-3′), type I collagen (COL1A1; osteogenic marker) (forward, 5′-ACGTCCTGGTGAAGTTGGTC-3′; reverse, 5′-ACCAGGGAAGCCTCTCTCTC-3′), type X collagen (COL10A1; marker of hypertrophy) (forward, 5′-CCCTCTTGTTAGTGCCAACC-3′; reverse, 5′-AGATTCCAGTCCTTGGGTCA-3′), matrix metalloproteinase 13 (MMP13; marker of terminal differentiation) (forward, 5′-AATTTTCACTTTTGGCAATGA-3′; reverse, 5′-CAAATAATTTATGAAAAAGGGATGC-3′), runt-related transcription factor 2 (RUNX2; osteogenic marker) (forward, 5′-GCAGTTCCCAAGCATTTCAT-3′; reverse, 5′-CACTCTGGCTTTGGGAAGAG-3′), ALP (osteogenic marker) (forward, 5′-TGGAGCTTCAGAAGCTCAACACCA-3′; reverse, 5′-ATCTCGTTGTCTGAGTACCAGTCC-3′), β-catenin (mediator of the Wnt signaling pathway for osteoblast lineage differentiation) (forward, 5′-CAAGTGGGTGGTATAGAGG-3′; reverse, 5′-GCGGGACAAAGGGCAAGA-3′), and glyceraldehyde-3-phosphate dehydrogenase (housekeeping gene and internal control) (forward, 5′-GAAGGTGAAGGTCGGAGTC-3′; reverse, 5′-GAAGATGGTGATGGGATTTC-3′) (all 150 nM final concentration) [ 24, 32]. Control conditions included reactions using water and nonreverse-transcribed mRNA. Specificity of the products was confirmed by melting curve analysis and agarose gel electrophoresis. The threshold cycle (Ct) value for each gene of interest was measured for each amplified sample using MxPro QPCR software (Stratagene), and values were normalized to glyceraldehyde-3-phosphate dehydrogenase expression by using the 2^–ΔΔCt^ method, as described previously [ 24, 32].

### Statistical analyses

Each treatment condition was performed in triplicate in three independent experiments for each patient. Data are expressed as the mean ± standard deviation of separate experiments. The *t* test and the Mann–Whitney rank-sum test were used where appropriate. *P* < 0.05 was considered statistically significant.

## Results

### Sustained expression of IGF-I in undifferentiated hMSCs via rAAV gene transfer

**Figure 1:**
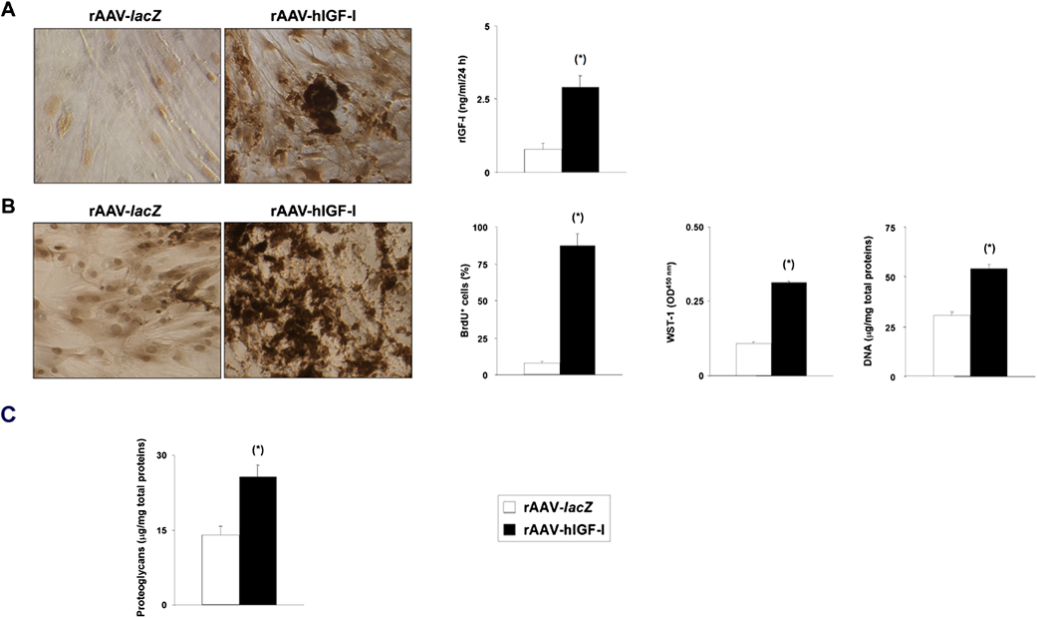
**Recombinant adeno-associated virus-mediated IGF-I gene transfer in undifferentiated monolayer cultures of human bone marrow-derived mesenchymal stem cells.** Cells were transduced with rAAV-hIGF-I or rAAV-*lacZ* (20 μl each vector) as described in Methods and histologically processed after 21 days to monitor **(A)** transgene (insulin-like growth factor I) expression by immunocytochemical analysis and enzyme-linked immunosorbent assay, **(B)** levels of cell proliferation by immunocytochemical detection of bromodeoxyuridine (BrdU) incorporation and histomorphometry, by WST-1 assay, and by detection of the DNA contents, and **(C)** levels of proteoglycan synthesis (magnification × 4). *Statistically significant compared with rAAV-*lacZ*. hIGF-I, human insulin-like growth factor I; OD, optical density; rAAV, recombinant adeno-associated virus.

hMSCs were first transduced with the candidate rAAV-hIGF-I vector in undifferentiated monolayer cultures compared with a control condition (application of a reporter rAAV-*lacZ* gene vector) [ 24, 32] to examine the ability of rAAV to mediate overexpression of the growth factor over time in these cells *in vitro* at an undifferentiated stage. Sustained, intense immunoreactivity to IGF-I was detected in cells transduced with rAAV-hIGF-I compared with the control treatment after 5 days (data not shown) and for up to 21 days, with transduction efficiencies of 76 to 84% (Figure 1A). This finding was corroborated by an analysis of the IGF-I production levels in IGF-I-transduced cells (1.2 ± 0.2 vs. 0.3 ± 0.1 ng/ml/24 hours in *lacZ*-treated cells on day 14, and 2.9 ± 0.4 vs. 0.8 ± 0.2 ng/ml/24 hours on day 21; an up to fourfold difference, always *P* ≤ 0.001) (Figure 1A).

### Effects of rAAV-hIGF-I transduction on the biological activities of undifferentiated hMSCs

The candidate rAAV-hIGF-I vector was next applied to undifferentiated monolayer cultures of hMSCs to investigate the effects of IGF-I treatment on the biological activities of the cells (proliferation, matrix synthesis) compared with control *lacZ* treatment. Immunodetection of BrdU incorporation in transduced cells revealed significantly increased levels of proliferation mediated by rAAV-hIGF-I compared with rAAV-*lacZ* (82 to 93% vs. ≤9% BrdU^+^ cells on day 21; up to 10.3-fold difference, *P* ≤ 0.001) (Figure 1B). These results were corroborated by the results of a WST-1 assay (~0.313 and 0.110 optical density at 450 nm in IGF-I-treated vs. *lacZ*-treated cells on day 21; 2.8-fold difference, *P* ≤ 0.001) (Figure 1B) and when monitoring the DNA contents in the cultures (53.9 ± 2.3 and 30.6 ± 1.8 μg/mg total proteins in IGF-I-treated vs. *lacZ*-treated cells on day 21; 1.8-fold difference, *P* ≤ 0.001) (Figure 1B). Further analyses also revealed that application of rAAV-hIGF-I significantly increased the proteoglycan contents in the cultures at the end of the evaluation period compared with *lacZ* treatment (25.7 ± 2.3 vs. 14.1 ± 1.7 μg/mg total proteins on day 21; 1.8-fold difference, *P* ≤ 0.001) (Figure 1C).

### Prolonged rAAV-mediated IGF-I expression in chondrogenically differentiated hMSCs

**Figure 2:**
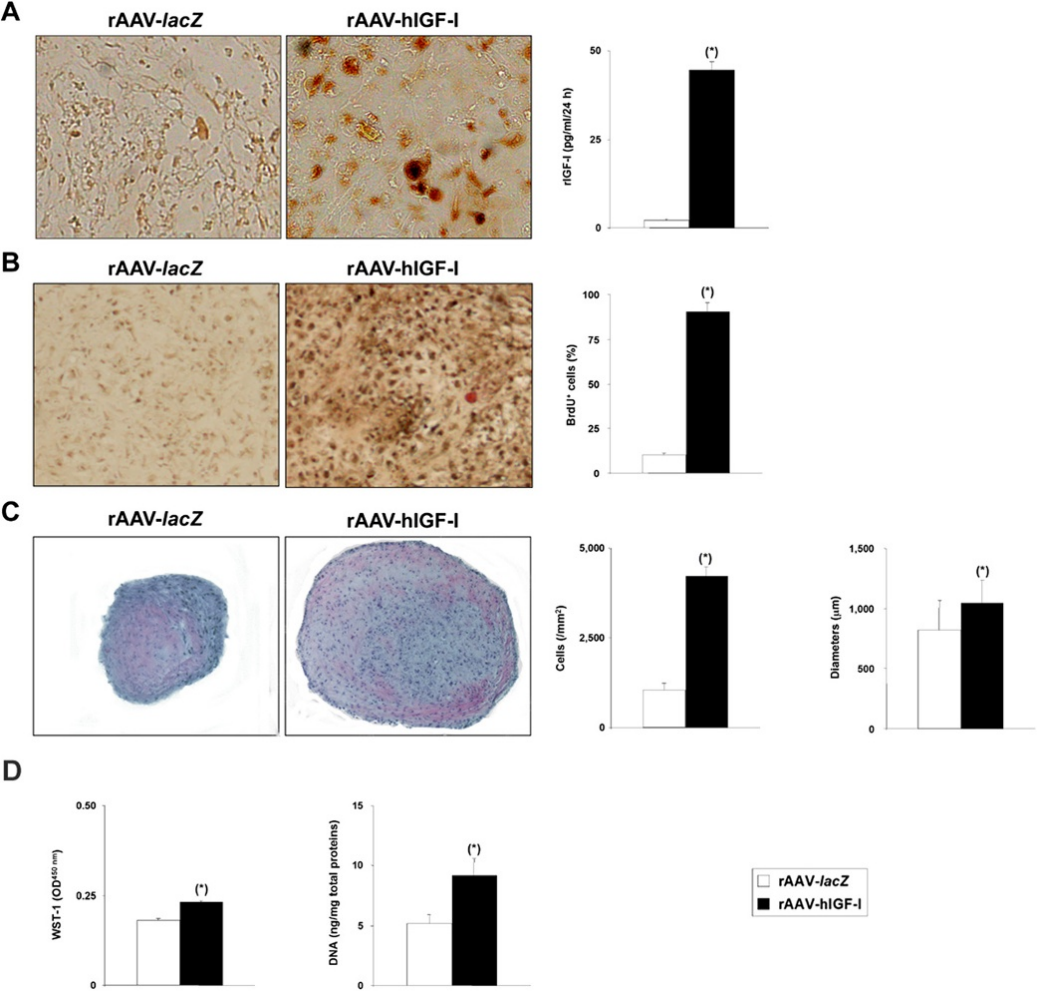
**Recombinant adeno-associated virus-mediated IGF-I gene transfer in chondrogenically induced cultures of human bone marrow-derived mesenchymal stem cells.** Human bone marrow-derived mesenchymal stem cell aggregates were transduced with rAAV-hIGF-I or rAAV-*lacZ* (40 μl each vector) as described in Methods and were histologically processed after 21 days to monitor **(A)** transgene (insulin-like growth factor I) expression by immunohistochemical analysis (magnification × 20) and enzyme-linked immunosorbent assay, and the levels of cell proliferation by immunohistochemical detection of bromodeoxyuridine (BrdU) incorporation and histomorphometry **(B)** (magnification × 10), by hematoxylin and eosin staining and histomorphometry **(C)** (magnification × 4), or by biochemical assays (WST-1 assay and detection of the DNA contents) **(D)**. *Statistically significant compared with rAAV-*lacZ*. hIGF-I, human insulin-like growth factor I; OD, optical density; rAAV, recombinant adeno-associated virus.

hMSCs were then transduced with rAAV-hIGF-I in chondrogenically induced aggregate cultures [ 10, 12, 13], again using rAAV-*lacZ* as a control condition because rAAV does not impair the potency of these cells [ 20, 24, 32, 39], in order to evaluate the ability of the candidate vector to promote the expression of IGF-I over time in cells committed toward the chondrocyte phenotype, with a focus on day 21 where chondrogenesis is known to occur [ 10, 12, 13]. In agreement with the findings in monolayer cultures, a strong, durable signal specific for the growth factor was detected by immunohistochemistry in cells transduced with rAAV-hIGF-I compared with *lacZ* for up to 21 days, with transduction efficiencies of 74 to 81% (Figure 2A). This result was also substantiated by an analysis of the IGF-I production levels in IGF-I-transduced cells (44.5 ± 2.3 vs. 2.1 ± 0.4 pg/ml/24 hours in *lacZ*-treated aggregates or 1.9 ± 0.2 pg/ml/24 hours in untreated aggregates on day 21; 21.2-fold to 23.4-fold difference, *P* ≤ 0.001, while there was no difference between controls, *P* = 0.130) (Figure 2A).

### Effects of rAAV-hIGF-I treatment on the biological activities and differentiation potential of chondrogenically induced hMSCs

Cells were next transduced with rAAV-hIGF-I and induced in aggregate cultures toward chondrogenesis in order to examine the effects of IGF-I treatment upon the biological activities and chondrogenic differentiation potential of the cells versus the *lacZ* control condition. As we and others clearly reported previously that rAAV gene transfer does not impair the potency of hMSCs [ 20, 24, 32, 39], we did not further include a condition without vector treatment. Immunodetection of BrdU incorporation in IGF-I-transduced cells revealed significantly increased levels of proliferation compared with *lacZ* (87 to 94% vs. ≤11% BrdU^+^ cells on day 21; up to 8.5-fold difference, *P* ≤ 0.001) (Figure 2B). These results were confirmed by an analysis of the cell densities on hematoxylin and eosin-stained sections from aggregates (4,220 ± 254 vs. 1,050 ± 187 cells/mm^2^ on day 21; fourfold difference, *P* ≤ 0.001) (Figure 2C), by the results of a WST-1 assay (~0.231 and 0.182 optical density at 450 nm in IGF-I-treated vs. *lacZ*-treated aggregates on day 21; 1.3-fold difference, *P* ≤ 0.001) (Figure 2D), or when monitoring the DNA contents in the cultures (9.2 ± 1.4 and 5.2 ± 0.7 ng/mg total proteins in IGF-I-treated vs. *lacZ*-treated aggregates on day 21; 1.8-fold difference, *P* ≤ 0.001) (Figure 2D). Also of note, application of the IGF-I vector significantly increased the diameters of the aggregates at the end of the period of evaluation compared with *lacZ* transduction (1,044 ± 190 vs. 822 ± 244 μm; 1.3-fold difference, *P* = 0.021) (Figure 2C).

**Figure 3:**
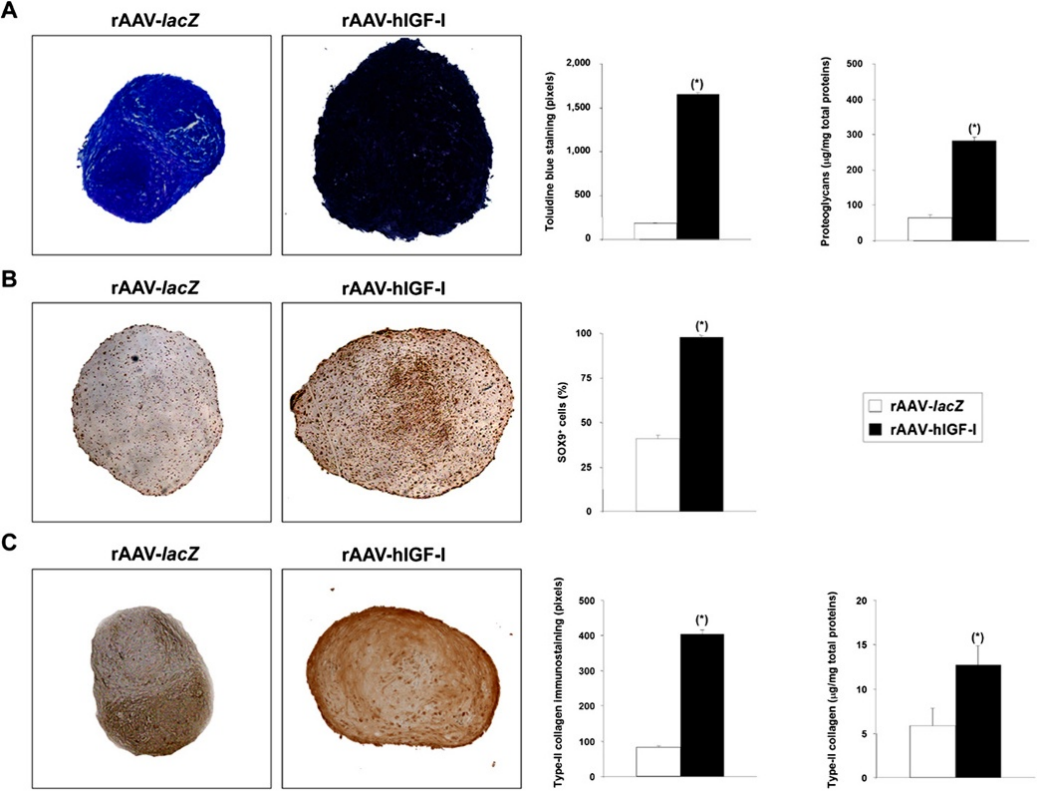
**Metabolic and differentiation activities in chondrogenically induced cultures of human bone marrow-derived mesenchymal stem cells transduced with rAAV-hIGF-I.** Human bone marrow-derived mesenchymal stem cell aggregates were transduced with rAAV-hIGF-I or rAAV-*lacZ* as described in Figure 2 and histologically processed after 21 days to evaluate the production of matrix proteoglycans (toluidine blue staining with histomorphometry and detection of the proteoglycan contents) **(A)** and the expression of SOX9 with histomorphometry **(B)** and type II collagen (specific immunodetection with histomorphometry and detection of the type II collagen contents) **(C)** (all at magnification × 4). *Statistically significant compared with rAAV-*lacZ*. hIGF-I, human insulin-like growth factor I; rAAV, recombinant adeno-associated virus.

**Figure 4:**
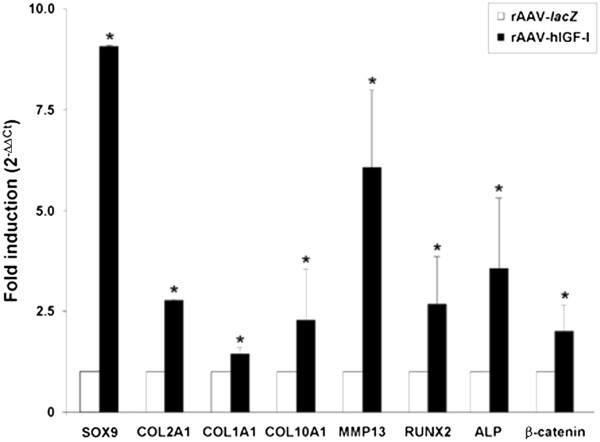
**Expression analyses in chondrogenically induced cultures of human bone marrow-derived mesenchymal stem cells transduced with rAAV-hIGF-I.** Human bone marrow-derived mesenchymal stem cell aggregates were transduced with rAAV-hIGF-I or rAAV-*lacZ* as described in Figure 2 and processed on day 21 for gene expression analysis by real-time reverse transcription-polymerase chain reaction amplification after total cellular RNA extraction and cDNA synthesis, as described in Methods. The genes analyzed included the transcription factor SOX9, type II, type I, and type X collagen (COL2A1, COL1A1, COL10A1), matrix metalloproteinase 13 (MMP13), the transcription factor RUNX2, alkaline phosphatase (ALP), and β-catenin, with glyceraldehyde-3-phosphate dehydrogenase (GAPDH) serving as a housekeeping gene and internal control (primers are listed in Methods). Threshold cycle (Ct) values were obtained for each target and GAPDH as a control for normalization, and fold inductions (relative to *lacZ*-treated aggregates) were measured using the 2^–ΔΔCt^ method. *Statistically significant compared with rAAV-*lacZ*. hIGF-I, human insulin-like growth factor I; rAAV, recombinant adeno-associated virus.

When samples were processed to monitor the metabolic and differentiation activities of hMSCs, successful chondrogenesis was noted on histological sections of aggregates as evidenced by toluidine blue staining and type II collagen deposition, revealing more intense staining when the IGF-I vector was applied (1,657 ± 22 vs. 181 ± 8 and 405 ± 12 vs. 83 ± 4 pixels of toluidine blue staining and type II collagen immunostaining, respectively, in IGF-I-treated vs. *lacZ*-treated aggregates on day 21; up to 9.2-fold difference, always *P* ≤ 0.001) (Figure 3A,C). These results were substantiated by an evaluation of the proteoglycan and type II collagen contents in the aggregates (283.5 ± 10.5 and 64.5 ± 7.8 μg proteoglycans/mg total proteins and 12.7 ± 2.2 and 5.9 ± 1.9 μg type II collagen/mg total proteins in IGF-I-treated vs. *lacZ*-treated aggregates on day 21; up to 4.4-fold difference, always *P* ≤ 0.001) (Figure 3A,C), possibly due to an increase in SOX9 expression promoted by IGF-I gene transfer (98 ± 1% vs. 41 ± 2% of SOX9^+^ cells, respectively, in IGF-I-treated vs. *lacZ*-treated aggregates on day 21; 2.4-fold difference, *P* ≤ 0.001) (Figure 3B). An analysis of the gene expression profiles by real-time reverse transcription (RT)-PCR further confirmed these findings (9.1-fold and 2.8-fold higher SOX9 and COL2A1 expression levels, respectively, in IGF-I-treated vs. *lacZ*-treated aggregates, always *P* ≤ 0.001) (Figure 4).

**Figure 5:**
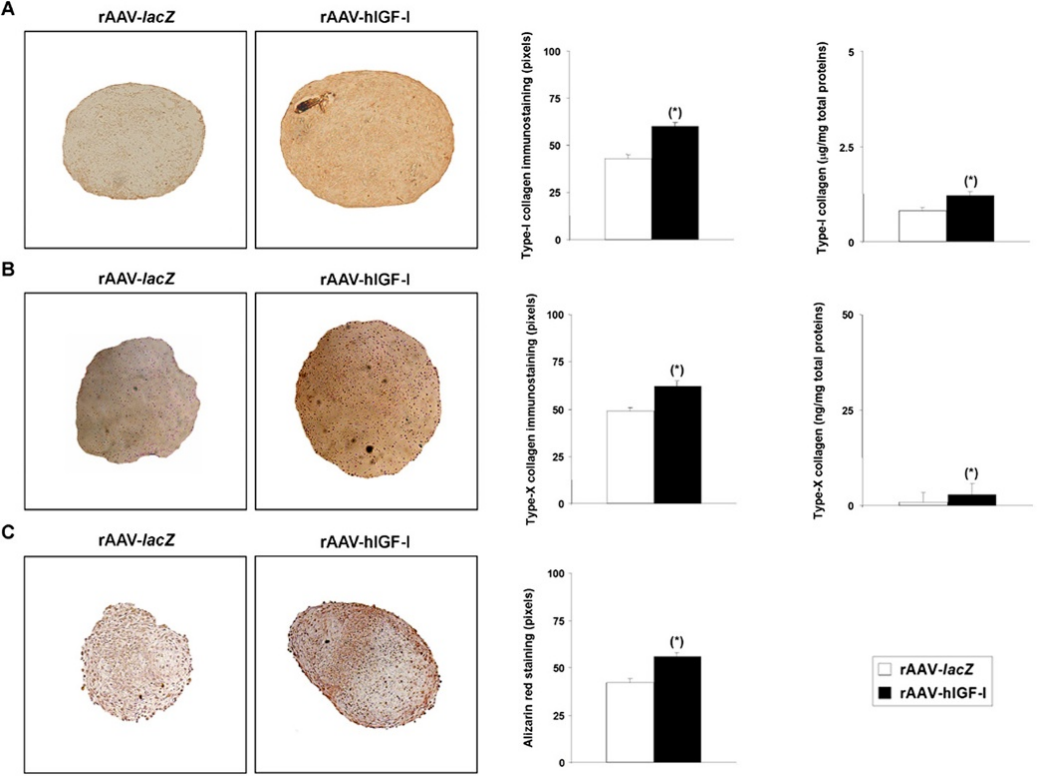
**Hypertrophic differentiation in chondrogenically induced cultures of human bone marrow-derived mesenchymal stem cells transduced with rAAV-hIGF-I.** Human bone marrow-derived mesenchymal stem cell aggregates were transduced with rAAV-hIGF-I or rAAV-*lacZ* as described in Figure 2 and histologically processed after 21 days to examine the expression of type I collagen **(A)** and type X collagen **(B)** by immunohistochemistry/histomorphometry and by analysis of the type I collagen **(A)** and type X collagen **(B)** contents, and to evaluate matrix mineralization (alizarin red staining with histomorphometry) **(C)** (all at magnification × 4). *Statistically significant compared with rAAV-*lacZ*. hIGF-I, human insulin-like growth factor I; rAAV, recombinant adeno-associated virus.

Interestingly, administration of the IGF-I vector compared with *lacZ* led to increases in the intensity of type I and type X collagen immunostaining and of alizarin red staining (60 ± 2 vs. 43 ± 2, 62 ± 3 vs. 49 ± 2, and 56 ± 2 vs. 42 ± 2 pixels of type I and type X collagen immunostaining and alizarin red staining, respectively, in IGF-I-treated vs. *lacZ*-treated aggregates on day 21; up to 1.4-fold difference, always *P* ≤ 0.001) (Figure 5A,B,C). These findings were corroborated by an evaluation of the type I and type X collagen contents in the aggregates (36.3 ± 2.9 and 22.8 ± 2.7 μg type I collagen/mg total proteins and 1.2 ± 0.1 and 0.8 ± 0.1 ng type X collagen/mg total proteins in IGF-I-treated vs. *lacZ*-treated cells on day 21; up to 1.6-fold difference, always *P* ≤ 0.001) (Figure 5A,B) and by real-time RT-PCR analysis (1.4-fold and 2.2-fold higher COL1A1 and COL10A1 expression levels, respectively, in IGF-I-treated vs. *lacZ*-treated aggregates; always *P* ≤ 0.001) (Figure 4). Of further note, such an analysis also revealed enhanced expression profiles for MMP13, RUNX2, ALP, and β-catenin upon IGF-I gene transfer compared with *lacZ* (6.1-fold, 2.5-fold, 3.6-fold, 1.9-fold, and 1.9-fold, respectively; always *P* ≤ 0.001) (Figure 4).

### Effects of rAAV-hIGF-I treatment on the osteogenic and adipogenic differentiation potential of hMSCs

**Figure 6:**
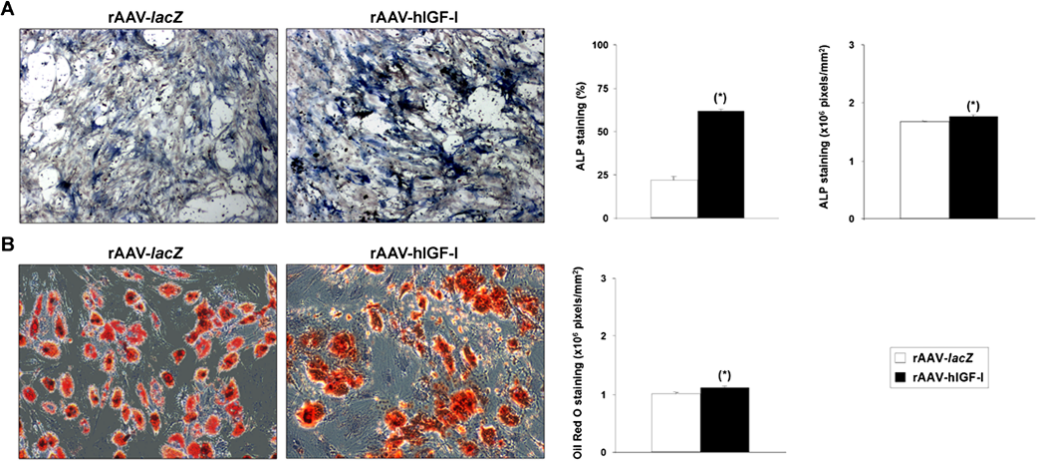
**Analyses in osteogenically and adipogenically differentiated cultures of human bone marrow-derived mesenchymal stem cells transduced with rAAV-hIGF-I.** Cells in monolayer cultures were transduced with rAAV-hIGF-I or rAAV-*lacZ* (40 μl each vector) and induced toward osteogenic or adipogenic differentiation as described in Methods. Cultures were processed on day 21 for **(A)** alkaline phosphatase (ALP) staining with histomorphometry (osteogenesis; magnification × 4) and **(B)** Oil Red O staining with histomorphometry (adipogenesis; magnification × 10). *Statistically significant compared with rAAV-*lacZ*. hIGF-I, human insulin-like growth factor I; rAAV, recombinant adeno-associated virus.

The candidate IGF-I vector was next provided to osteogenically and adipogenically differentiated hMSCs over time to estimate further the effects of the growth factor via rAAV application on the potential for osteogenic and adipogenic differentiation of the cells compared with control *lacZ* treatment. Successful osteogenic differentiation was noted in the induced cultures as evidenced by ALP staining (Figure 6A). Notably, application of rAAV-hIGF-I significantly increased the percentage of stained areas as well as the intensities of staining after 21 days compared with *lacZ* condition (62 ± 1% vs. 22 ± 2% and 1.773 ± 0.017 vs. 1.678 ± 0.009 × 10^6^ pixels/mm^2^, respectively; up to 1.1-fold difference, always *P* ≤ 0.001) (Figure 6A). Successful adipogenic differentiation was also achieved in the induced cultures as seen by the accumulation of lipid droplets after staining with Oil Red O (Figure 6B). Interestingly, while there was no significant difference between the percentages of stained areas on day 21 between IGF-I-treated and *lacZ*-treated samples (75 ± 1% and 74 ± 1%, *P* = 0.215), the intensities of staining were significantly higher when the rAAV-hIGF-I was applied (1.110 ± 0.001 vs. 1.009 ± 0.002 × 10^6^ pixels/mm^2^; 1.1-fold difference, *P* ≤ 0.001) (Figure 6B).

## Discussion

The concept of transplanting progenitor cells such as MSCs in articular cartilage defects is a promising approach to enhance the intrinsic healing capacities of this particular tissue [ 40– 42] and is already a clinical reality employed to treat patients [ 11]. Yet the production of an original, hyaline cartilage tissue has never be reported in any of the lesions so far treated with stem cells, leading instead to the formation of a fibrocartilage with poor biomechanical properties, showing the need for improved procedures. In this regard, the genetic manipulation of MSCs is an attractive approach to address such an issue by improving their potentiality for enhanced cartilage repair processes upon transplantation in the defects. Here, we focused on delivering a candidate sequence coding for the anabolic and mitogenic IGF-I factor by transduction with rAAV, one of the safest and most effective gene vehicles available to date for human gene therapy, in light of our previous work showing that such a construct allows for stable reconstruction of human osteoarthritic cartilage *in situ* [ 36].

Our data first demonstrate that highly effective and prolonged expression of IGF-I can be achieved upon rAAV transduction in hMSCs using undifferentiated and chondrogenically induced cultures for at least 21 days (the longest time point evaluated), with transduction efficiencies reaching up to 84%, all in good agreement with previous findings using the same vector to target articular chondrocytes [ 36] or when applying other rAAV to this cell type [ 20, 24, 32, 39], probably due to the good persistence of rAAV transgenes in their targets [ 43]. The levels achieved here in hMSC aggregates with rAAV were 44.5 pg IGF-I/ml/24 hours after 21 days of culture, while higher but only very short-term production has been reported upon transduction with IGF-I adenoviral vectors (from 75 to 30 ng/ml in hMSCs between days 3 and 7, and of 50 to 80 ng/ml for only 3 days in animal cells, with undetectable expression levels beyond these time points) [ 19, 44, 45]. This is probably due to the much higher MOIs applied in these previous studies (MOI = 100 to 250) whereas we used much lower rAAV MOIs (MOI = 4, 25-fold to 63-fold less vector) in a similar three-dimensional environment, all in good agreement with the properties of each class of vector [ 46, 47].

The data further indicate that IGF-I overexpression using rAAV in the conditions applied here was capable of stimulating both the proliferative and anabolic activities of hMSCs in undifferentiated monolayer cultures over a prolonged period of time (21 days), probably due to the sustained levels of IGF-I expression achieved with this stable class of gene transfer vector, all in good agreement with the properties of the growth factor [ 36, 48, 49]. Most remarkably, treatment with the current rAAV IGF-I construct also allowed one to enhance the differentiation of these cells in chondrogenically induced aggregate cultures compared with the control (*lacZ*) treatment, as noted by the increased expression levels and deposition of proteoglycans and type II collagen. These results are in marked contrast with previous findings from different groups who reported that application of IGF-I to MSCs via gene transfer was not capable of inducing such processes in similar culture conditions [ 19, 44, 45]. It is important to mention that all these earlier studies focused on the use of much less efficient adenoviral vectors that allowed only for very short-term transgene expression (not beyond a week), while we evidenced a prolonged production of IGF-I with rAAV over the whole period of evaluation (21 days). Moreover, our data are strongly supported by work from Uebersax and colleagues [ 50], who showed that the slow, sustained release of IGF-I from biocompatible scaffolds can promote the chondrogenic differentiation of hMSCs over a similar, extended period of time. Of further note, we observed that rAAV IGF-I gene transfer was capable of stimulating the expression levels of SOX9, a key chondrogenic factor for MSC differentiation, as noted when providing the same vector to human chondrocytes [ 36] and in good agreement with previous findings [ 51], probably resulting in the enhanced commitment of the cells toward chondrogenesis compared with control treatment. Interestingly, we noted that administration of rAAV IGF-I led to the expression of hypertrophic and osteogenic markers in hMSCs. This was probably a result of the enhanced expression levels for MMP13 (marker of terminal differentiation), RUNX2 (transcription factor controlling the osteoblastic expression of COL1, COL10, and MMP13), ALP (osteogenic marker), and β-catenin (mediator of the Wnt pathway for osteogenic differentiation) in response to IGF-I treatment, concordant with the osteogenic activities of the growth factor further evidenced here and with previous findings [ 52]. Also of note, IGF-I gene transfer with rAAV enhanced the adipogenic differentiation of the cells, again in good agreement with previous findings [ 53].

Overall, these results suggest that a tight control of IGF-I production is a prerequisite for the optimal use of the current rAAV vector in ongoing, clinically relevant stem cell-based experimental models of cartilage defects *in vivo* [ 54, 55]. A balance of expression that allows one to produce sufficient levels of IGF-I for chondrogenic differentiation without activation of premature hypertrophic maturation and ossification may be reached by applying various (lower) vector doses, using lineage-specific/tissue-specific or regulatable promoters, and/or by providing additional factors that have the ability to prevent such undesirable effects. Among them, FGF-2, the SOX transcription factors, and molecules that may silence osteogenic pathways such as an anti-Runx2/Cbfa1 small interfering RNA are potent candidates to achieve this goal as they have been already reported for their ability to delay hypertrophic processes in MSCs [ 24, 29, 30, 32, 34]. In this regard, rAAV vectors are strong tools that can promote a simultaneous expression of separate genes in target cells without interference [ 56]. In conclusion, the present findings show the potential of using rAAV-mediated gene transfer as a tool for future stem cell-based approaches to treat articular cartilage defects.

## Conclusions

In summary, our study suggests that the genetic modification of hMSCs via rAAV for the durable expression of IGF-I is a strong tool to induce the chondrogenic commitment of the cells but that a tight regulation of the production levels will be necessary to adapt the strategy in experimental settings of articular cartilage repair to control the premature terminal differentiation of the cells *in vivo*.

## Abbreviations

ALP: alkaline phosphatase
BrdU: bromodeoxyuridine
DMEM: Dulbecco’s modified Eagle’s medium
ELISA: enzyme-linked immunosorbent assay
FGF-2: basic fibroblast growth factor
hIGF-I: human insulin-like growth factor I
hMSC: human bone marrow-derived mesenchymal stem cell
IGF-I: insulin-like growth factor I
MMP13: matrix metalloproteinase 13
MOI: multiplicity of infection
MSC: bone marrow-derived mesenchymal stem cell
PCR: polymerase chain reaction
rAAV: recombinant adeno-associated virus
RT: reverse transcription
RUNX2: runt-related transcription factor 2
TGFβ: transforming growth factor beta.
